# Myocardial creep and cardiorespiratory motion correction improves diagnostic accuracy of Rubidium-82 cardiac positron emission tomography

**DOI:** 10.1007/s12350-023-03360-x

**Published:** 2023-08-25

**Authors:** Martin Lyngby Lassen, Thomas Rasmussen, Christina Byrne, Lene Holmvang, Andreas Kjaer, Philip Hasbak

**Affiliations:** 1 Department of Clinical Physiology, Nuclear Medicine and PET, University Hospital Copenhagen–Rigshospitalet, Copenhagen, Denmark; 2 Cluster for Molecular Imaging, Department of Biomedical Sciences, Faculty of Health and Medical Sciences, University of Copenhagen, Copenhagen, Denmark; 3 Department of Cardiology, The Heart Centre, Rigshospitalet, Copenhagen, Denmark

**Keywords:** Ischemic heart disease, motion correction, cardiac PET, myocardial perfusion imaging, Rubidium-82, Cardiopatía isquémica, corrección de movimiento, PE cardíaco, imágenes de perfusión miocárdica, Rubidio-82, Maladie cardiaque ischémique, correction de mouvement, TEP cardiaque, étude de perfusion myocardique, Rubidium-82

## Abstract

**Aim:**

To evaluate the feasibility of retrospectively detecting and correcting periodical (cardiac and respiratory motion) and non-periodical shifts of the myocardial position (myocardial creep) using only the acquired Rubidium-82 positron emission tomography raw (listmode) data.

**Methods:**

This study comprised 25 healthy participants (median age = 23 years) who underwent repeat rest/adenosine stress Rubidium-82 myocardial perfusion imaging (MPI) and 53 patients (median age = 64 years) considered for revascularization who underwent a single MPI session. All subjects were evaluated for myocardial creep during MPI by assessing the myocardial position every 200 ms. A proposed motion correction protocol, including corrections for cardiorespiratory and creep motion (3xMC), was compared to a guideline-recommended protocol (Standard_Recon_). For the volunteers, we report test-retest repeatability using standard error of measurements (SEM). For the patient cohort, we evaluated the area under the receiver operating curve (AUC) for both stress and ischemic total perfusion deficits (sTPD and iTPD, respectively) using myocardial ischemia defined as fractional flow reserve values < 0.8 in the relevant coronary segment as the gold standard.

**Results:**

Test-retest repeatability was significantly improved following corrections for myocardial creep (SEM; sTPD: Standard_Recon_ = 2.2, 3xMC = 1.8; iTPD: Standard_Recon_ = 1.6, 3xMC = 1.2). AUC analysis of the ROC curves revealed significant improvements for iTPD measurements following 3xMC [sTPD: Standard_Recon_ = 0.88, 3xMC = 0.92 (*P* = .21); iTPD: Standard_Recon_ = 0.88, 3xMC = 0.95 (*P* = .039)].

**Conclusion:**

3xMC has the potential to improve the diagnostic accuracy of myocardial MPI obtained from positron emission tomography. Therefore, its use should be considered both in clinical routine and large-scale multicenter studies.

**Supplementary Information:**

The online version contains supplementary material available at 10.1007/s12350-023-03360-x.

## Spanish Abstract

**Objetivo:**

Evaluar la factibilidad de detectar y corregir retrospectivamente los cambios periódicos (movimiento cardíaco y respiratorio) y no periódicos de la posición del miocardio (deslizamiento del miocardio) utilizando solo los datos en crudo sin procesar (modo de lista) de la tomografía por emisión de positrones con Rubidio-82.

**Metodología:**

Este estudio contó con 25 participantes sanos (mediana de edad = 23 años) que se sometieron a imágenes repetidas de perfusión miocárdica (MPI) con Rubidio-82 en protocolo reposo/estrés con adenosina y 53 pacientes (mediana de edad = 64 años) considerados para revascularización que se sometieron a un solo estudio de MPI. A todos los sujetos se les evaluó el deslizamiento del miocardio durante el MPI mediante la evaluación de su posición cada 200 ms. Un protocolo de corrección de movimiento propuesto, que incluye correcciones para movimiento cardiorrespiratorio y de deslizamiento (3xMC), se comparó con un protocolo recomendado por las guías (StandardRecon). Para los voluntarios, informamos la repetibilidad test-retest utilizando medidas estándar de error (SEM). Para la cohorte de pacientes, evaluamos el área bajo la curva operativa del receptor (AUC) tanto para el estrés como para los déficits de perfusión total isquémicos (sTPD y iTPD, respectivamente) usando isquemia miocárdica definida como valores de reserva de flujo fraccional < 0.8 en el segmento coronario relevante como el estándar dorado de referencia.

**Resultados:**

La repetibilidad test-retest mejoró significativamente después de las correcciones para el deslizamiento del miocardio (SEM; sTPD: StandardRecon = 2.2, 3xMC = 1.8; iTPD: StandardRecon = 1.6, 3xMC = 1.2). El análisis AUC de las curvas ROC reveló mejoras significativas para las mediciones de iTPD después de 3xMC (sTPD: StandardRecon = 0.88, 3xMC = 0.92 (*P* = .21); iTPD: StandardRecon = 0.88, 3xMC = 0.95 (*P* = .039)).

**Conclusión:**

3xMC tiene el potencial de mejorar la precisión diagnóstica del MPI miocárdico obtenido a partir de la tomografía por emisión de positrones. Por lo tanto, su uso debe ser considerado tanto en los estudios clínicos rutinarios como en estudios multicéntricos a gran escala.

## Chinese Abstract

**目的:**

本研究旨在评估使用铷-82正电子发射扫描获得的原始数据, 回顾性检测和校正心肌位置的周期性 (心脏和呼吸运动) 和非周期性变化 (心肌蠕变) 的可行性。

**方法:**

这项研究包括25名接受静息/腺苷负荷铷-82心肌灌注显像MPI的健康参与者 (中位年龄 = 23岁) ; 以及53名仅接受一次MPI拟行血运重建的患者 (中位年龄=64岁) 。我们通过每隔200毫秒评估心肌位置的手段来评估MPI期间的心肌蠕变。本研究比较包括心肺和蠕变运动校正的运动校正方案(3xMC) 与指南推荐的方案(Standard_Recon_) 。在健康参与者队列中, 我们使用标准测量误差SEM来报告重测信度。在患者队列中, 我们评估了负荷和缺血总灌注缺损(sTPD和iTPD) 的接收者操作特征曲线下面积AUC。心肌缺血的金标准被定义为有关冠状动脉段的血流储备分数< 0.8。

**结果:**

校正心肌蠕变后, 重测信度显著提高(SEM: sTPD: Standard_Recon_ = 2.2, 3xMC = 1.8; iTPD: Standard_Recon_ = 1.6, 3xMC = 1.2) 。AUC分析表明, 在3xMC校正后, iTPD的测量准确性显著提高(sTPD: Standard_Recon_ = 0.88, 3xMC = 0.92(*P* = .21) ; iTPD: Standard_Recon_ = 0.88, 3xMC = 0.95(*P* = .039) ) 。

**结论:**

运动矫正方案3xMC可提高PET-MPI诊断的准确性。因此, 在临床常规检查和大规模多中心研究中应考虑应用该方案。

## French Abstract

**Titre:**

La correction pour le mouvement cardio-respiratoire et le glissement myocardique améliore la précision diagnostique de la tomographie par émission de positrons cardiaque au Rubidium-82.

**But:**

Évaluer la faisabilité de détecter rétrospectivement et de corriger les changements périodiques (mouvements cardiaques et respiratoires) et non-périodiques de la position du myocarde (glissement myocardique) en utilisant seulement les données brutes (en mode liste) de la tomographie par positrons au Rubidium-82.

**Méthodologie:**

L’étude incluait 25 volontaires sains (âge médian = 23 ans) qui ont eu des études répétées de perfusion myocardique (ÉPM) au Rubidium-82 repos-adénosine et 53 patients (âge médian = 64 ans) considérés pour revascularisation qui ont eu une seule séance de ÉPM. Tous les sujets ont été étudiés pour le glissement myocardique durant ÉPM en évaluant la position du myocarde chaque 200ms. Un protocole de correction de mouvement proposé, incluant les corrections pour les mouvements cardio-respiratoires et de glissement (3xMC), a été comparé au protocole recommandé par les lignes directrices (Recon.Standard). Pour les volontaires, nous rapportons la répétabilité test-retest en utilisant l’erreur standard des mesures (ESM). Pour la cohorte de patients, nous avons évalué la zone dérivée de la courbe de la fonction d’efficacité du receveur (ROC) pour les déficits à l’effort et les déficits de perfusion ischémiques totaux (respectivement sTPD et iTPD) en utilisant l’ischémie myocardique définie comme ayant une valeur de réserve de flot fractionnel < 0.8 dans un segment coronarien pertinent comme mesure étalon.

**Résultats:**

La répétabilité test-retest était améliorée de façon significative après les corrections pour le glissement myocardique (ESM;sTPD : Recon.Standard = 2.2, 3xMC = 1.8, iTPD : Recon.Standard = 1.6, 3xMC = 1.2). L’analyse de la zone sous la ROC a révélé des améliorations significatives pour les mesures de iTPD après le 3xMC (sTPD : Recon.Standard = 0.88, 3xMC = 0.92 (*P* = .21) : iTPD : Recon.Standard = 0.88, 3xMC = 0.95 (*P* = .039)).

**Conclusions:**

Le 3xMC a le potentiel d’améliorer la précision diagnostique des ÉPM obtenues à partir de la tomographie par émission de positrons. Par conséquent, son utilisation devrait être considérée à la fois en routine clinique mais aussi dans des études multicentriques à grande échelle.

## Introduction

Rubidium-82 (^82^Rb) is the most commonly used radiotracer in positron emission tomography (PET) myocardial perfusion imaging (MPI).^1,2^ ^82^Rb permits quantification of both rest and stress MPI, with the latter being obtained using pharmacological stressing agents such as adenosine. Intravenous administration of adenosine is used to achieve conditions of stable hyperemia in the heart; unfortunately, the hyperemia elevates the already high risks of patient motion, and non-periodical repositioning of the heart in the mediastinum, also known as myocardial creep.^1,3^ Although myocardial creep occurs frequently in cardiac MPI, only a few correction techniques have been proposed.^3–5^ Common for the proposed correction techniques is that they apply to dynamic reconstructions employed for myocardial blood flow assessments and only permit corrections for inter-frame motion.^3–5^ While introducing an averaged image of the later frames in the motion-corrected, dynamic image series may offer a solution, the resulting images may have increased noise and residual blur introduced by the missing intra-frame motion correction. Therefore, this study aimed to evaluate (a) the feasibility of retrospectively extracting information on myocardial creep using only ^82^Rb-PET raw data (listmode data) and (b) the impact of motion correction of the myocardial creep events. To correct the myocardial creep, we performed triple-gated reconstructions, employing a dual-gated cardiorespiratory (cardiac contraction and respiratory motion) reconstruction protocol for each detected myocardial creep event, followed by image co-registration; thus, obtaining a triple-motion-corrected image series (3xMC).^6,7^ The results of ^82^Rb-PET MPI were compared to fractional flow reserve (FFR) as a gold standard reference for coronary intervention.

## Materials and methods

### Study population

We included two study populations, one comprising 25 healthy volunteers and the other 53 patients with intermediate to high pre-test likelihood of cardiovascular disease with no known coronary artery disease (CAD) (n = 30) or known CAD (n = 23) referred for both ^82^Rb-PET and subsequent coronary angiography and FFR assessments. Both study populations gave both informed and written consent to participate in the study. The studies were filed under protocol numbers [H-15009293 and H-42014046, respectively], following approval from the Scientific Ethics Committee and the Capital Region of Denmark, and the Danish Data Protection Agency.

The volunteer cohort comprised 25 healthy young persons who underwent repeat ^82^Rb rest/adenosine stress MPI within 2 weeks (Table 1).^8,9^ Inclusion criteria were age > 18 years, no regular consumption of medicine, no known medical condition, no use of tobacco and euphoric substances (except alcohol) within 3 months before study participation, and no caffeine intake (plasma caffeine concentrations < 1,000 µg/L). Exclusion criteria were pregnancy, allergy or intolerance to theophylline or adenosine, any prior medical history of asthma, and inability to adhere to the study protocol.

**Table 1:** Volunteer and patient demographics

	Volunteer cohort (n = 25)	Patients with no known CAD (n = 30)	Patients with known CAD (N = 23)
Age (years)^a^	22 [22; 23]	64 [58;70]	66 [58; 71]
Gender, females, N (%)	11 (44%)	6 (20%)	6 (26%)
BMI (kg/m^2^)^a^	22 [21; 24]	28 [24; 33]	29 [26; 30]
Single/two/three-vessel disease	0/0/0	5/3/5	0/10/13
Rest LVEF /stress LVEF^a^	63 [59; 67]/72 [66; 75]	67 [58; 71]/69 [62; 75]	55 [41; 62]/58 [41; 63]
Current smoking, N (%)	9 (36%)	6 (20%)	2 (9%)
COPD, N (%)	0 (0%)	4 (13%)	5 (22%)
Diabetes mellitus, N (%)	0 (0%)	6 (20%)	5 (22%)
Hypertension, N (%)	0 (0%)	18 (60%)	1 (4%)
Hypercholesterolemia, N (%)	0 (0%)	21 (70%)	20 (87%)
Family history of IHD, N (%)	Unknown	12 (40%)	12 (52%)
History of myocardial infarction	0 (0%)	1 (3%)	9 (39%)
Previous PCI/CABG	0 (0%)	0 (0%)/0(0%)	7 (30%)/4(17%)

*BMI*, body mass index; *COPD*, chronic obstructive pulmonary disease; *ACE-inhibitors*, angiotensin-converting enzyme inhibitors; *AT-II inhibitors*, angiotensin II receptor blockers

^a^Median and interquartile range

The patient cohort included 53 patients (Table 1).

Inclusion criteria were age > 50 years, while exclusion criteria were claustrophobia, severe asthma, or renal failure (plasma creatinine > 140 µM). Coronary stenoses > 50% identified by invasive angiography were, in all cases, assessed for hemodynamic significance by measuring FFR during continuous adenosine infusion (140 μg/kg/min) for 2 minutes. A FFR< 80% was considered significant and led to subsequent percutaneous coronary intervention (PCI) where technically possible.

### Imaging protocol

All study participants underwent rest/adenosine stress MPI in a 128 slice Siemens Biograph mCT PET system using targeted injection doses of 1,100 MBq (30 mCi) ^82^Rb. Pharmacological stressing was obtained using adenosine infused intravenously at a rate of 140 mg/kg/min for 6 minutes, with PET emission acquisition starting 2.5 minutes into the infusion. Each imaging session started with a low-dose CT for attenuation correction purposes acquired using a free-breathing protocol,^10^ followed by the PET emission scans. All participants were instructed to abstain from caffeine at least 16 hours before each of the imaging sessions.

### Image reconstruction

Two PET reconstruction protocols were evaluated. Both reconstruction protocols employed data obtained between 150 and 360 seconds into the ^82^Rb-PET acquisitions,^11^ using the following reconstruction parameters: 2 iterations, 21 subsets, corrections for Time-of-Flight and point spread, and a 5 mm Gaussian filtration. The first reconstruction protocol provided a conventional static reconstruction (Standard_Recon_), as used in routine assessments. The second reconstruction protocol provided a triple-motion-corrected image series (3xMC), including corrections for myocardial creep and cardiorespiratory motion correction (described in detail below).

### Myocardial creep and respiratory motion detection

**Figure 1:**
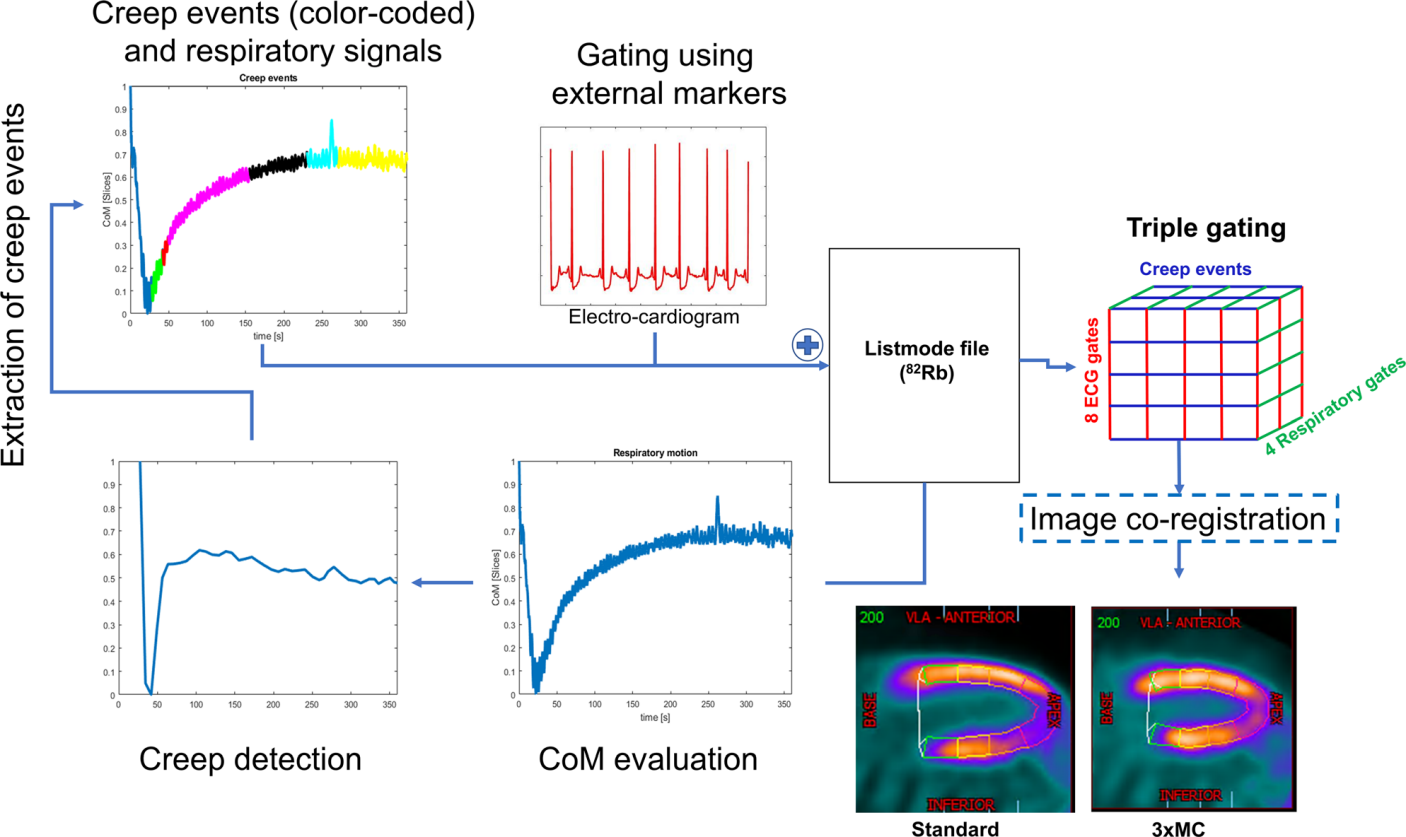
Data-driven motion detection and correction. All datasets obtained ECG-triggering events from a 3-lead external marker, while myocardial creep and respiratory motion were detected using data-driven techniques employing only the acquired ^82^Rb-PET raw data. The myocardial position was calculated with a temporal resolution of 200 ms (CoM evaluation). The creep detection was obtained comparing the count rates observed in the myocardium to the count rates obtained in the surrounding tissues, using a convolution function. Changes in the signal of more than 20% within 5 seconds (sudden creep event) or 8% over 30 seconds (drifting creep event) were considered myocardial creep events. The respiratory signal was extracted by filtering the CoM signal with a frequency between 0.1 and 0.65 Hz). Using the triggering signals, the data was reconstructed into a triple gating event which was co-registered to obtain the 3xMC. *CoM*, center-of-mass

The myocardial creep events were detected retrospectively in the acquired PET raw data (listmode) using a center-of-mass-based analysis,^6,7,12^ employing a temporal resolution of 200 ms to facilitate the detection of myocardial creep events with a high temporal resolution. This proposed motion detection technique differed from previous attempts at detecting patient repositioning events^6,7^ as ^82^Rb MPI scans are affected by tracer-kinetics during the acquisitions. To this end, we hypothesize that the myocardial creep events may be detected by comparing radiotracer kinetic uptake in tissues with and without specific uptake. For the myocardium, ^82^Rb is being trapped via Na-K-ATPase, while healthy lung tissue, on the other hand, tends to have a non-specific uptake pattern. Given both tissues follow the same respiratory repositioning events, any significant change in the center-of-mass for the two tissues indicates a myocardial creep event. In this study, we tested this assumption by convolving the center-of-mass signals obtained in the myocardium and surrounding tissues (Figure 1). From the convoluted center-of-mass signals, it is possible to extract information on myocardial creep using a piece-wise fit [myocardial creep was defined as changes of ≥ 20% within 5 seconds (repositioning event) or 8% over 30 seconds (change in the respiratory baseline)], while the respiratory signal was obtained by filtering all the inspiratory peaks in the frequency-band (0.1-0.65 Hz).

### Myocardial creep and respiratory motion correction

3xMC was obtained by introducing a cardiorespiratory (dual-gated) reconstruction protocol for each myocardial creep event, as based on a previous proposal.^6^ All gated reconstructions were co-registered using a local-registration matrix surrounding the segmented heart ± 15 slices (slice thickness = 2.07 mm) in all directions). The co-registration was obtained using a non-rigid registration protocol (demons) in MatLab.

### Image analysis

We report the average translation observed in the myocardium during the scans, the total perfusion deficits for all reconstructed datasets [rest and stress total perfusion deficits (rTPD and sTPD, respectively)], and the ischemic total perfusion deficit (iTPD) calculated as the respective sTPD minus rTPD for the individual MPI sessions.^11,13^ Of note, rTPD, sTPD, and iTPD are given in % of the left ventricular wall volume. In this study, an iTPD ≥ 10% was considered abnormal.^14^

We also report the test-retest repeatability using the standard error of measurement (SEM) of the motion-induced perfusion deficits observed for the healthy volunteers. For the patient cohort, we report the crosstab assessments, including the prevalence of disease, the sensitivities, and specificities, in addition to the positive and negative predictive values obtained for the Standard_Recon_ and 3xMC assessments.

In addition, two experienced readers (combined experience of > 30 years) were asked to visually evaluate the images concerning the potential of motion-induced artifacts in the images while being blinded to the used reconstruction protocol. All assessments were performed in the clinical software toolbox (QPET, Cedars-Sinai). The images were scored on a scale from 1 to 5, with 1 being scored when the readers were certain of motion-induced artifacts in the automatic assessments; conversely, 5 was given when the images did not show any signs of motion artifacts. Finally, we report the TPD values and the area under the receiver operating curve (AUC) for the patient cohort as the primary end-point for this study.^11,13^

### Statistical analysis

The data were tested for normality using Shapiro-Wilk test. Continuous data were presented as mean ± SD or median and interquartile ranges, and categorical data as percentages. Employing the FFR findings for the patient population, the impact of the myocardial creep motion correction was evaluated using receiver operating characteristic curves. All receiver operating characteristic curves were compared using the DeLong and DeLong method,^15^ using the area under the curve (AUC) as the primary end-point for this study. Test-retest repeatability was calculated as the standard error of measurement (SEM) and the 95% confidence intervals of the SEM. Differences in bias were evaluated using the Pitman-Morgan test, with *P*-values < .05 being considered statistically significant. Finally, the number of creep events and patient motion across the different cohorts were evaluated using a two-way ANOVA.

## Results

Baseline characteristics for the volunteer and patient groups are shown in Table 1. For the patient population, 21 of the 53 had significant myocardial ischemia [as observed for the Standard_Recon_ (^82^Rb-PET iTPD > 10%)], whereas 25 patients had FFR < 0.8 in at least 1 coronary artery and underwent subsequent PCI (n = 22) or CABG (n = 3) intervention.

### Motion during the scans

All study participants had a minimum of one myocardial creep event during the PET acquisition, with a median of 3 (range: 1-7) myocardial creep events detected in the volunteer group and 2 (range: 1-5) observed in the patient cohort (Table 2). The myocardial creep events moved the heart by as much as 26.1 mm (volunteers: 23.2 mm, patients: 26.1 mm), of which > 47% was observed in the scanners *z*-direction [volunteers: 11.1 mm (47.8%), patients (16.8 mm (64.3%)]. Including cardiorespiratory motion correction, the heart moved up to 33.9 mm (volunteers: 25.5 mm, patients: 33.9 mm), of which motion in *z*-direction accounted for > 52% of the motion [volunteers: 13.4 mm (52.5%), patients: 20.6 (60.7%)] (Table 2). On average, the number of creep events and magnitude of myocardial motion was significantly higher in the volunteers than in the patients (Table 2). Visual assessments of the images revealed that the 3xMC images were scored, on average, to have reduced motion-induced artifacts in the images (Table 3).

**Table 2:** Number of detected myocardial creep events and the corresponding motion observed during the stress MPI sessions (The corresponding numbers for the rest scans can be found in supplementary material 1)

	Volunteers	Patients
Number of myocardial creep events^a^	3 [1; 7]	2 [1; 5]* (*P* < .001)
*Z*-motion, mm^b^	9.1 ± 3.4	7.0 ± 4.3* (*P* = .006)
3D motion, mm^bc^	14.3 ± 5.2 (64.6 ± 8.7%)	11.5 ± 0.33* (59.9 ± 11.6%) (*P* = .011)

Of note, * denotes significant differences in the volunteer and patient cohorts

*3xMC*, triple-motion-corrected (myocardial creep, respiratory motion and cardiac contraction corrected)

^a^Median and full range

^b^Mean ± SD

^c^Numbers in parenthesis indicate the % wise contribution of motion in the systems *z*-direction

**Table 3:** Image quality and sTPD and iTPD values observed in the patient cohort before and after 3xMC

	Rest scan		Stress scan		Reserve	
	Standard_Recon_	3xMC	Standard_Recon_	3xMC	Standard_Recon_	3xMC
Image quality before and after 3xMC						
Visual score^a^	3.1 ± 0.5	3.5 ± 0.6	2.8 ± 0.5	3.4 ± 0.7	N/A	N/A
Average TPD values before and after 3xMC						
TPD^a^	4.6 ± 6.4	5.5 ± 7.2	15.3 ± 14.2	16.4 ± 16.0	10.7 ± 10.9	16.5 ± 11.2

Of note, the image quality shows the average of two independent readers with a combined experience of > 30 years

^a^Mean ± SD

### Test-retest repeatability (volunteer group)

Test-retest repeatability was calculated for the volunteer group. The 3xMC datasets had reduced SEM and bias compared to the Standard_Recon_ protocol (Table 4). Of note, the 3xMC images had significantly reduced bias compared to the Standard_Recon_ reconstructions.

**Table 4:** Test-retest repeatability and bias observed for the young volunteers without cardiovascular disease

	rTPD		sTPD		iTPD	
	SEM	Bias	SEM	Bias	SEM	Bias
Standard_Recon_^a^	0.9 [0; 3.1]	1.4	2.2 [0; 6.6]	2.3	1.6 [0; 4.5]	1.5
3xMC^a^	0.7 [0; 1.8]	0.4* (*P* = .004)	1.8 [0; 4.5]	1.0* (*P* = .033)	1.2 [0; 3.0]	0.6* (*P* = .039)

Of note, the bias was calculated as the average sTPD/iTPD obtained for the respective reconstructions, as no findings were expected. The 95% confidence interval is the brackets. Of note, * marks significant differences in the bias between the motion-corrected images and the standard reconstructions (*P* < .05)

*SEM*, standard error of measurement; *rTPD*, rest total perfusion deficit; *sTPD*, stress total perfusion deficit; *iTPD*, ischemic total perfusion deficit

^a^Mean [95% confidence interval]

### Stress and ischemic perfusion analysis (patient group)

**Figure 2:**
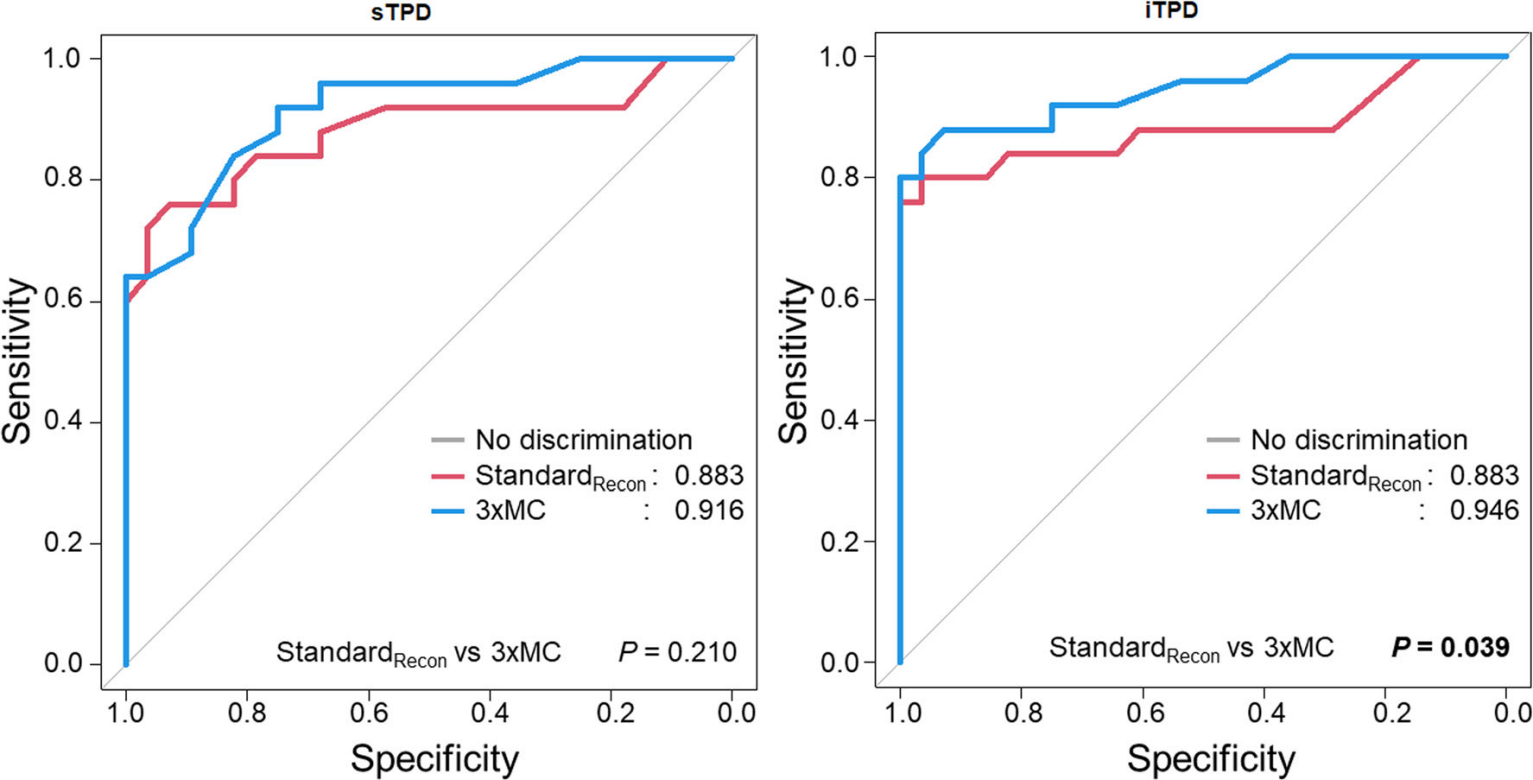
sTPD, and iTPD analysis of the 53 patients with outcome measures. 3xMC significantly increased the AUC for the iTPD assessments. *sTPD*, stress total perfusion deficit, *iTPD*, ischemic total perfusion deficit

**Figure 3:**
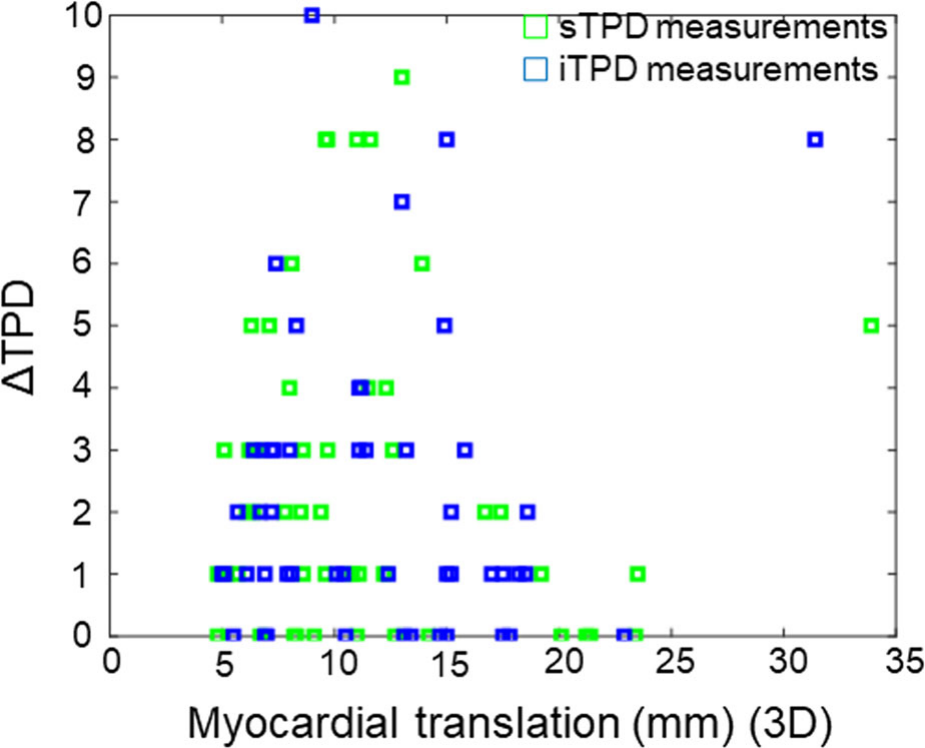
Changes in sTPD and iTPD assessment as a function of myocardial translations (3D-motion observed) during the acquisition. Trends between absolute changes in sTPD and iTPD for standard and 3xMC reconstructions and motion asserted on the myocardium are observed for the patient cohort. Of note, the myocardial translation for the iTPD is calculated as the mean translation of the rest and stress MPIs. *3D*, three dimensional motion (*x*, *y* and *z* motion); *ΔsTPD*, absolute change in sTPD observed between standard and 3xMC reconstruction protocols; *ΔiTPD*, absolute change in iTPD observed between standard and 3xMC reconstruction protocols; *sTPD*, stress total perfusion deficit; *iTPD*, ischemic total perfusion deficit

**Figure 4:**
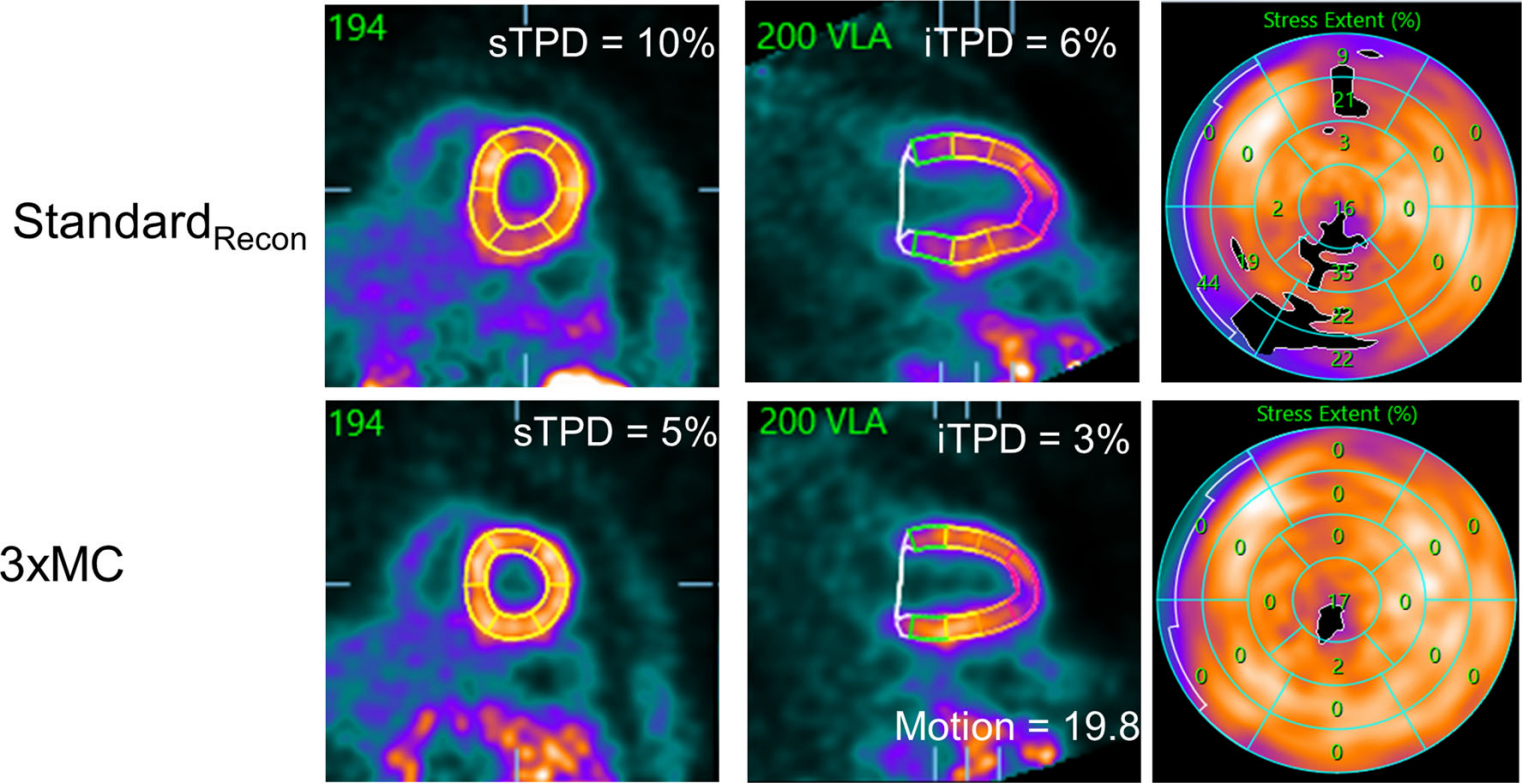
Case-example of a patient with motion-induced artifacts in the LAD and RCA territories. 3xMC removed the detrimental motion in the two vascular territories, and introduced a more homogenous activity distribution in the heart. A residual artifact, however, may be observed in the apical region. Subsequent showed no significant obstructive coronary artery stenoses. *sTPD*, stress total perfusion deficit; *iTPD*, ischemic total perfusion deficit; *3xMC*, triple-motion-corrected PET images (cardiorespiratory and creep corrected)

The diagnostic accuracy of the reconstruction protocols, where any FFR < 0.8 was considered the gold standard, was measured using AUC for both sTPD and iTPD. We report improvements in the AUC analysis for the 3xMC protocol for iTPD (sTPD: Standard_recon_ = 0.883, 3xMC = 0.916 (*P* = .210); iTPD: Standard_recon_ = 0.883, 3xMC = 0.946 (*P* = .039)) (Figure 2). 3xMC is reported to have significantly improved sensitivity compared to the assessment of Standard_Recon_, while the specificities were comparable (Sensitivity: Standard_Recon_ = 80.0%, 3xMC = 84.0% (*P* = .006), Specificity: Standard_Recon_ = 96.4%, 3xMC = 96.4%, *P* = .151) (Table 5). Of importance, the relative change in the sTPD and iTPD assessments indicated an association with the magnitude of motion observed during the stress MPI (Figure 3). Finally, the TPD values obtained for the 3xMC were elevated compared to the Standard_Recon_, although not significantly (Table 3). A case example of the impact of 3xMC is shown in Figure 4.

**Table 5:** Crosstab analysis of the data before and after 3xMC

	Standard_Recon_	3xMC
Prevalence of disease	47.2	47.2
Sensitivity	80.0	84.0
Specificity	96.4	96.4
Positive predictive value	95.2	95.5
Negative predictive value	84.4	87.1

Of note, all values are given in %

## Discussion

This study evaluated the impact of correcting for myocardial creep and cardiorespiratory (cardiac and respiratory) motion in two cohorts, one comprising young, healthy volunteers with repeat ^82^Rb MPI sessions within 2 weeks and the other patients considered for coronary intervention. The main finding of this study was improved test-retest repeatability in the volunteer cohort and improved AUC for the detection of adenosine-induced myocardial ischemia by FFR in the patient cohort (iTPD: *P* = .039) when employing the 3xMC. Likewise, a correlation trend was observed between motion during the MPI sessions and the relative change in the sTPD/iTPD assessments (Figure 3). Therefore, our results suggest that myocardial creep and cardiorespiratory motion correction significantly improve the accuracy of ^82^Rb MPI sessions and should be applied routinely.

In previous studies, cardiorespiratory and repositioning/myocardial creep events have been reported to have a detrimental impact on the assessment of both coronary plaques and myocardial perfusion.^3–6^ Despite the translatory impact of respiratory motion and myocardial creep, the clinical routine has a mainstay of motion-limiting ECG-gated reconstructions, which are used for assessments of the left ventricular ejection fraction.^16,17^ However, studies employing dual-correction protocols (cardiorespiratory) have been proposed with great success.^12,18^ In terms of myocardial creep corrections during MPI sessions, the primary solutions revolve around passive detections of myocardial creep during assessments of dynamic frames employed for myocardial blood flow analysis. Here, the passive correction only permits inter-frame motion correction and does not consider within-frame myocardial creep events, which might limit the effect of the myocardial creep correction technique.^3–5^ Further, this correction technique does not permit myocardial creep correction of perfusion assessments which often endures several minutes' data. Finally, this technique does not permit corrections for cardiorespiratory motion, which is known to affect quantitative accuracy.^12,18^ The proposed myocardial creep detection technique permits the detection of myocardial creep events dynamically during the acquisition and, at the same time, extracts information on the respiratory motion, which can be used for 3xMC corrections. The myocardial creep detection employed in this study expands on previous attempts at detecting patient repositioning events as tracer-kinetics influence the count rate-based assessments during the scans. In brief, the influence of tracer-kinetic on the count rate-based assessment was corrected by evaluating how the uptake pattern in the myocardium changed compared to the count rates observed in the surrounding lung tissues. This analysis permitted detection of the myocardial creep events, that occurs frequently (up to seven times) during the scans (Table 2). The myocardial creep and cardiorespiratory motion-induced myocardial translations of up 33.9 mm during the scan, with consequential changes in the sTPD and iTPD assessment of up to 10% to 12% (Figure 2). Of note, the improvements in the TPD assessments following 3xMC exceed the 2% improvement observed with the introduction of CT-attenuation correction in SPECT images; thus, indicating that the 3xMC has the potential to significantly better the prognostic and diagnostic assessments when evaluating ^82^Rb MPI.^19^ In general, the magnitude of motion observed during the scans reflected the change in sTPD and iTPD for patients who underwent revascularization following MPI (Figure 3).

In this study, the impact of myocardial creep was evaluated using two metrics, the test-retest repeatability of the sTPD and iTPD, obtained for the volunteers, and the AUC analyses obtained for the patients. Test-retest repeatability of the motion detection and correction technique is key for the clinical implementation; in this study, we tested the repeatability using SEM in the volunteer cohort in which no underlying perfusion deficits were expected. A more heterogeneous test-retest assessment of both sTPD and iTPD was observed when using Standard_Recon_ in comparison to the 3xMC technique, which in combination with an increased bias observed for the Standard_Recon_ highlights the detrimental impact of motion during rest/stress MPI sessions. The AUC assessments obtained for the elderly patient cohort with comorbidities support the finding in the healthy volunteer cohort. For the AUC assessments, the 3xMC provided significantly improved assessments of the ischemic burden (iTPD), highlighting the detrimental effect of motion during the MPI acquisitions (Figure 2). Further, the improved iTPD assessments obtained from 3xMC underline the importance of incorporating sophisticated motion correction protocols into routine assessments as the patients, on average, exert creep events 2.5 times during the 3.5 minutes reconstruction window considered in this study.

## Study limitations

This study had some limitations; the most important was the small cohort sizes evaluated in this study. Linked to the limitation, the small cohort of volunteers comprised healthy individuals who have no underlying comorbidities nor cardiovascular disease. Therefore, the findings for this cohort primarily consist of motion-induced perfusion deficits, likely introduced by the stressing agent. Hence, the test-retest repeatability is expected to be reflective of the actual underlying variation in the analysis. Although the patient cohort comprised only 53 patients, 25 showed positive FFR values and underwent subsequent revascularization. Therefore, the indications of the sTPD and iTPD assessments are assumed to reflect end-points obtained in bigger patient cohorts such as the Rubidium-ARMI.^20^ However, we acknowledge that the AUC might change slightly in bigger cohorts, though significant AUC improvements are still expected. Another limitation of the study is the time spent on performing the 3xMC, which is approximately 1.5 hours per creep event; thus, on average, 4.5 hours of reconstruction time for the typical patient analyzed in this study. We, however, are currently looking into shortening the time spent on the reconstructions using denoising alternatives.

## New knowledge gained

Myocardial creep is common during ^82^Rb MPI, with an average of 2-3 creep events occurring during the myocardial metabolism phase (150-360 seconds) in the MPI sessions. We report improved test-retest repeatability in the healthy volunteers and significantly improved AUC for both sTPD and iTPD analyses in the patients. Combined, these results highlight the need for 3xMC in routine ^82^Rb MPI assessments.

## Conclusions

We report significantly improved AUC for the iTPD assessments for ^82^Rb MPI sessions following 3xMC, compared to the Standard_Recon_ protocol. Further, the 3xMC improved the test-retest repeatability obtained in a cohort of healthy volunteers. Therefore, we propose to employ 3xMC in the clinical routine to strengthen the outcome measures obtained for patients undergoing routine ^82^Rb MPI sessions.

### Supplementary Information

Below is the link to the electronic supplementary material.

Supplementary file1 (DOCX 14 kb)

Supplementary file2 (PPTX 248 kb)

## Data Availability

The data is not publicly available.

## Abbreviations

3xMC: Triple-motion correction (myocardial creep and cardiorespiratory motion)
^82^Rb: Rubidium-82
AUC: The area under the receiver operating curve
FFR: Fractional flow reserve
iTPD: Ischemic total perfusion deficit
MPI: Myocardial perfusion imaging
PET: Positron emission tomography
rTPD: Rest total perfusion deficit
SEM: Using standard error of measurements
sTPD: Stress total perfusion deficit
