# Supplementary material for: Myocardial creep and cardiorespiratory motion correction improves diagnostic accuracy of Rubidium-82 cardiac positron emission tomography
Source: J Nucl Cardiol. 2023 Aug 25;30(6):2289–300. doi: 10.1007/s12350-023-03360-x (PMC10682154; doi:10.1007/s12350-023-03360-x)
Supplement: Supplementary file 1 — Supplementary file1 (DOCX 14 kb) [file 12350_2023_3360_MOESM1_ESM.docx]

**Myocardial creep and cardiorespiratory motion correction improves prognostic assessment of Rubidium-82 cardiac positron emission tomography**

Martin Lyngby Lassen, PhD^a^, Thomas Rasmussen, MD, PhD^a^, Christina Byrne, MD, PhD^a^, Lene Holmvang, MD, PhD^b^, Andreas Kjaer, MD, PhD, DMSc^a^, Philip Hasbak, MD, DMSc^a^

^a^Department of Clinical Physiology, Nuclear Medicine and PET & Cluster for Molecular Imaging, Copenhagen University Hospital - Rigshospitalet and University of Copenhagen

^b^Department of Cardiology, The heart centre, Rigshospitalet, Copenhagen, Denmark

Corresponding Author:
Martin Lyngby Lassen, PhD, Department of Clinical Physiology, Nuclear Medicine and PET and Cluster for Molecular Imaging, section 4011, Rigshospitalet and University of Copenhagen, Blegdamsvej 9, 2100 Copenhagen, Denmark

Tel: +45 35453520 Fax: +45 35454015

Email: [martin.lyngby.lassen@regionh.dk](mailto:)Total words: 3949

Short title: Triple-motion corrected perfusion assessment

Supplementary Table 1: Number of detected myocardial repositioning events and the corresponding motion observed during the rest MPI sessions measured in mm. Numbers in parenthesis indicate the % wise contribution of motion in the systems z-direction. Number of myocardial repositioning events is given in median [range], whereas motion is given in mm (mean±SD). * denotes significant differences between the two cohorts

|  | Volunteers | Patients |
| --- | --- | --- |
| Number of myocardial creep events^a^ | 3 [0; 8] | 1 [0; 4]* |
| 3xMC | | |
| Z-motion^b^ | 9.5±3.4 | 7.0±3.3* |
| 3D motion^bc^ | 15.2±4.5 (64.3±9.2%) | 12.2±5.8* (59.0±10.6%) |

^a^Median and full range

^b^Mean ±SD

^c^Numbers in parenthesis indicate the % wise contribution of motion in the systems z-direction.

3xMC = triple motion corrected (myocardial creep, respiratory motion and cardiac contraction corrected)
