# Supplementary material for: Myocardial creep and cardiorespiratory motion correction improves diagnostic accuracy of Rubidium-82 cardiac positron emission tomography
Source: J Nucl Cardiol. 2023 Aug 25;30(6):2289–300. doi: 10.1007/s12350-023-03360-x (PMC10682154; doi:10.1007/s12350-023-03360-x)
Supplement: Supplementary file 2 — Supplementary file2 (PPTX 248 kb) [file 12350_2023_3360_MOESM2_ESM.pptx]

## Slide 1
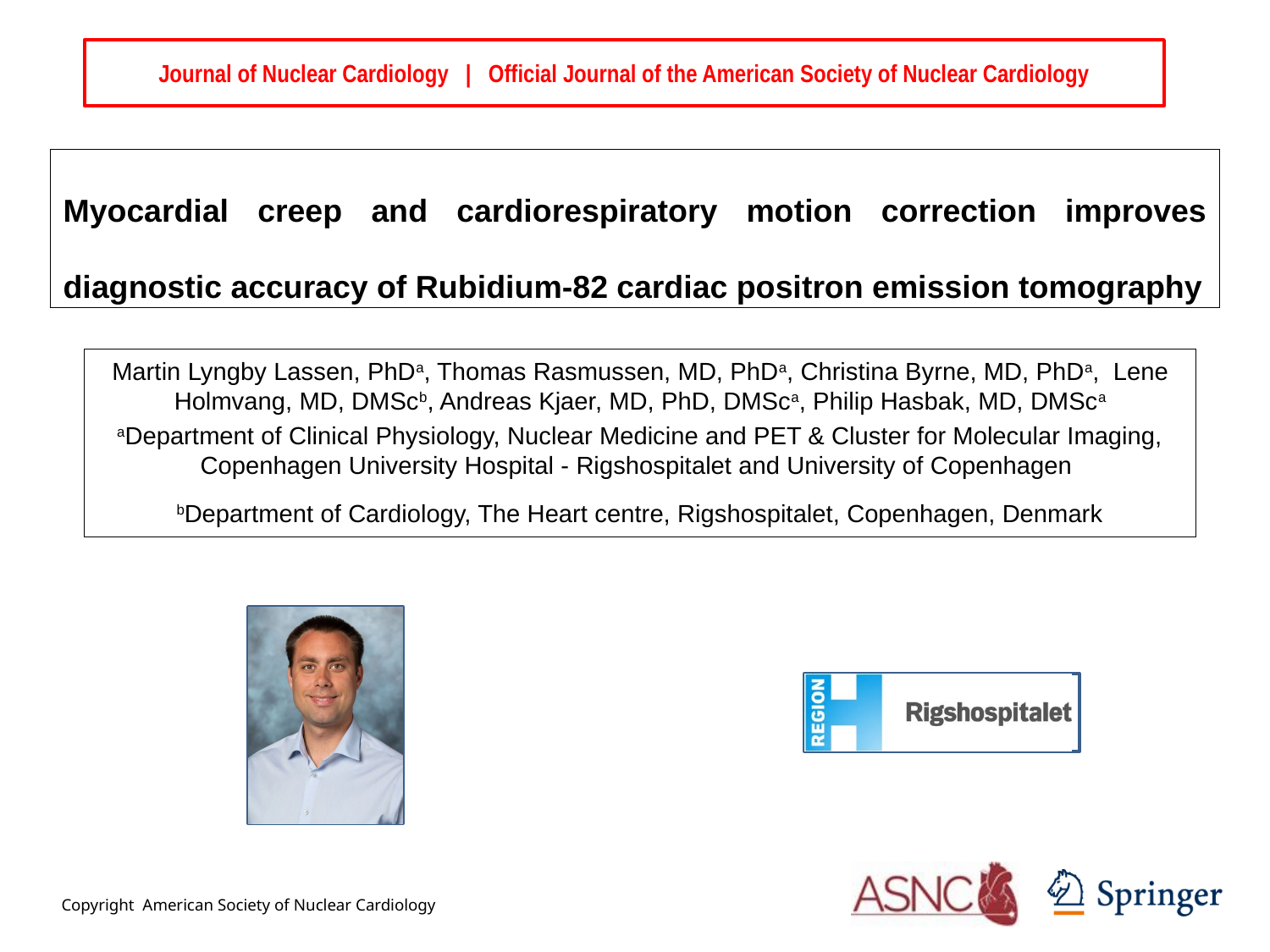

Journal of Nuclear Cardiology | Official Journal of the American Society of Nuclear Cardiology
# Myocardial creep and cardiorespiratory motion correction improves diagnostic accuracy of Rubidium-82 cardiac positron emission tomography
Martin Lyngby Lassen, PhDa, Thomas Rasmussen, MD, PhDa, Christina Byrne, MD, PhDa, Lene Holmvang, MD, DMScb, Andreas Kjaer, MD, PhD, DMSca, Philip Hasbak, MD, DMSca
aDepartment of Clinical Physiology, Nuclear Medicine and PET & Cluster for Molecular Imaging, Copenhagen University Hospital - Rigshospitalet and University of Copenhagen
bDepartment of Cardiology, The Heart centre, Rigshospitalet, Copenhagen, Denmark
Copyright American Society of Nuclear Cardiology

## Slide 2
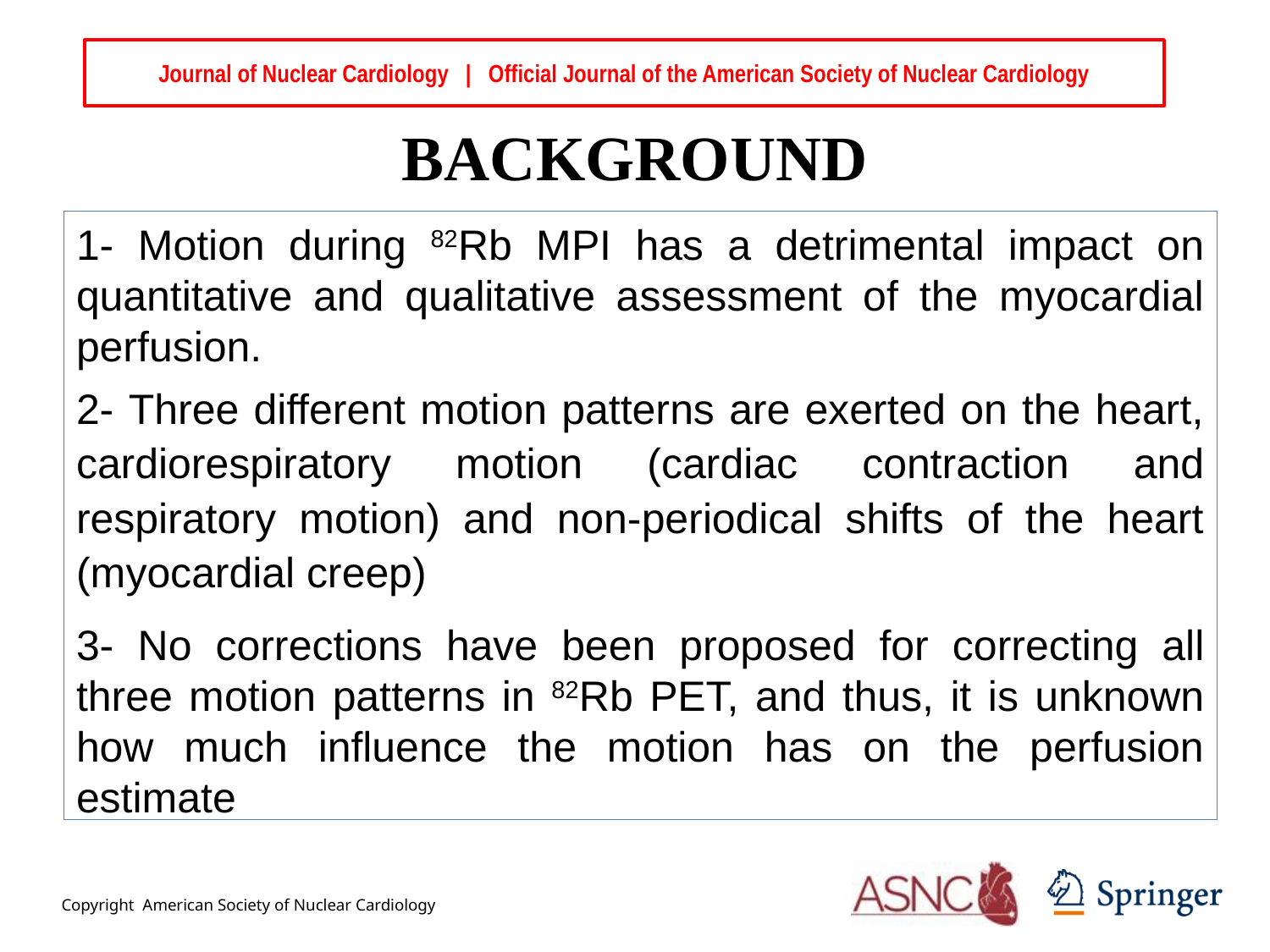

Journal of Nuclear Cardiology | Official Journal of the American Society of Nuclear Cardiology
# BACKGROUND
1- Motion during 82Rb MPI has a detrimental impact on quantitative and qualitative assessment of the myocardial perfusion.
2- Three different motion patterns are exerted on the heart, cardiorespiratory motion (cardiac contraction and respiratory motion) and non-periodical shifts of the heart (myocardial creep)
3- No corrections have been proposed for correcting all three motion patterns in 82Rb PET, and thus, it is unknown how much influence the motion has on the perfusion estimate
Copyright American Society of Nuclear Cardiology

## Slide 3
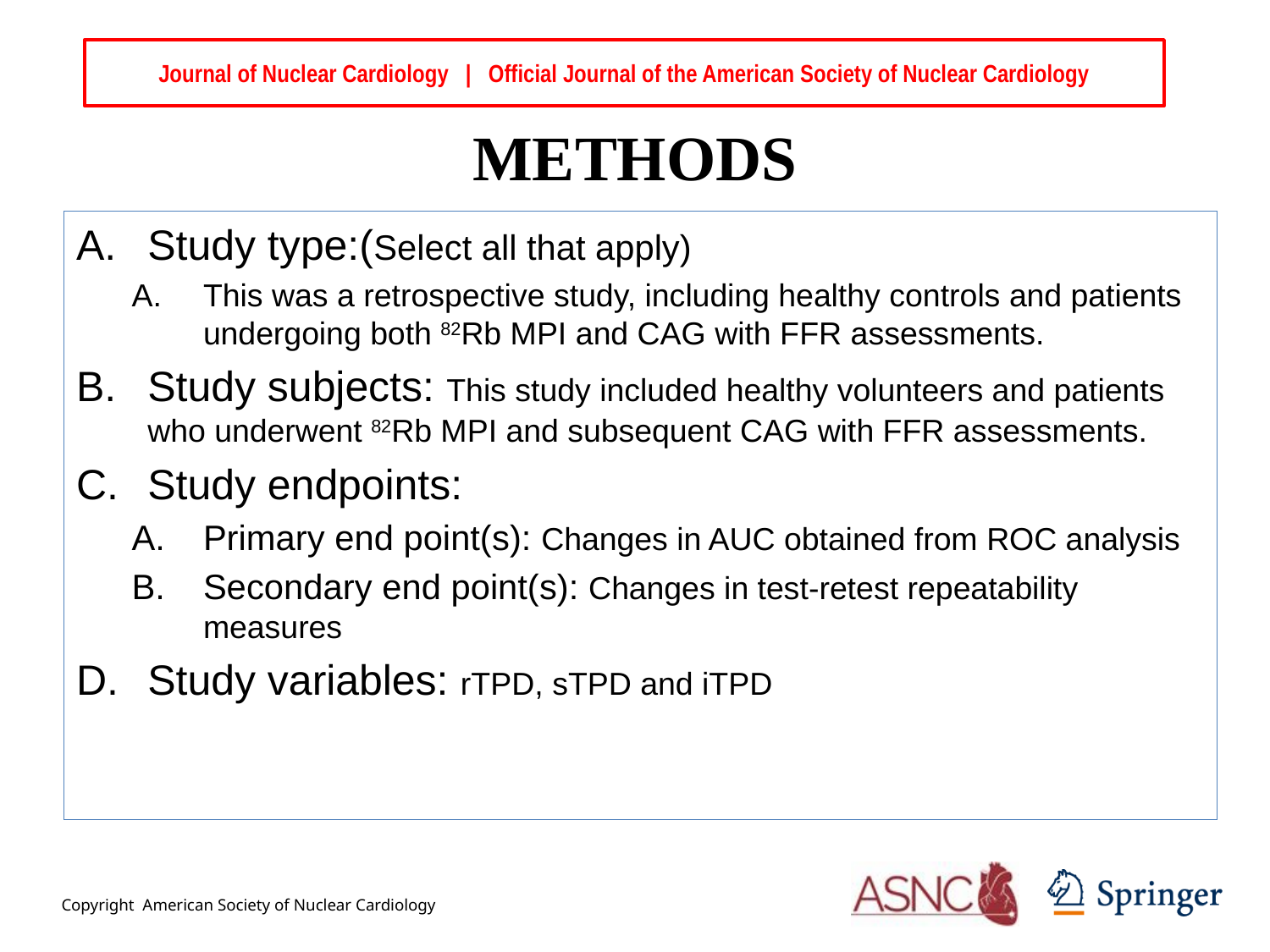

Journal of Nuclear Cardiology | Official Journal of the American Society of Nuclear Cardiology
# METHODS
Study type:(Select all that apply)
This was a retrospective study, including healthy controls and patients undergoing both 82Rb MPI and CAG with FFR assessments.
Study subjects: This study included healthy volunteers and patients who underwent 82Rb MPI and subsequent CAG with FFR assessments.
Study endpoints:
Primary end point(s): Changes in AUC obtained from ROC analysis
Secondary end point(s): Changes in test-retest repeatability measures
Study variables: rTPD, sTPD and iTPD
Copyright American Society of Nuclear Cardiology

## Slide 4
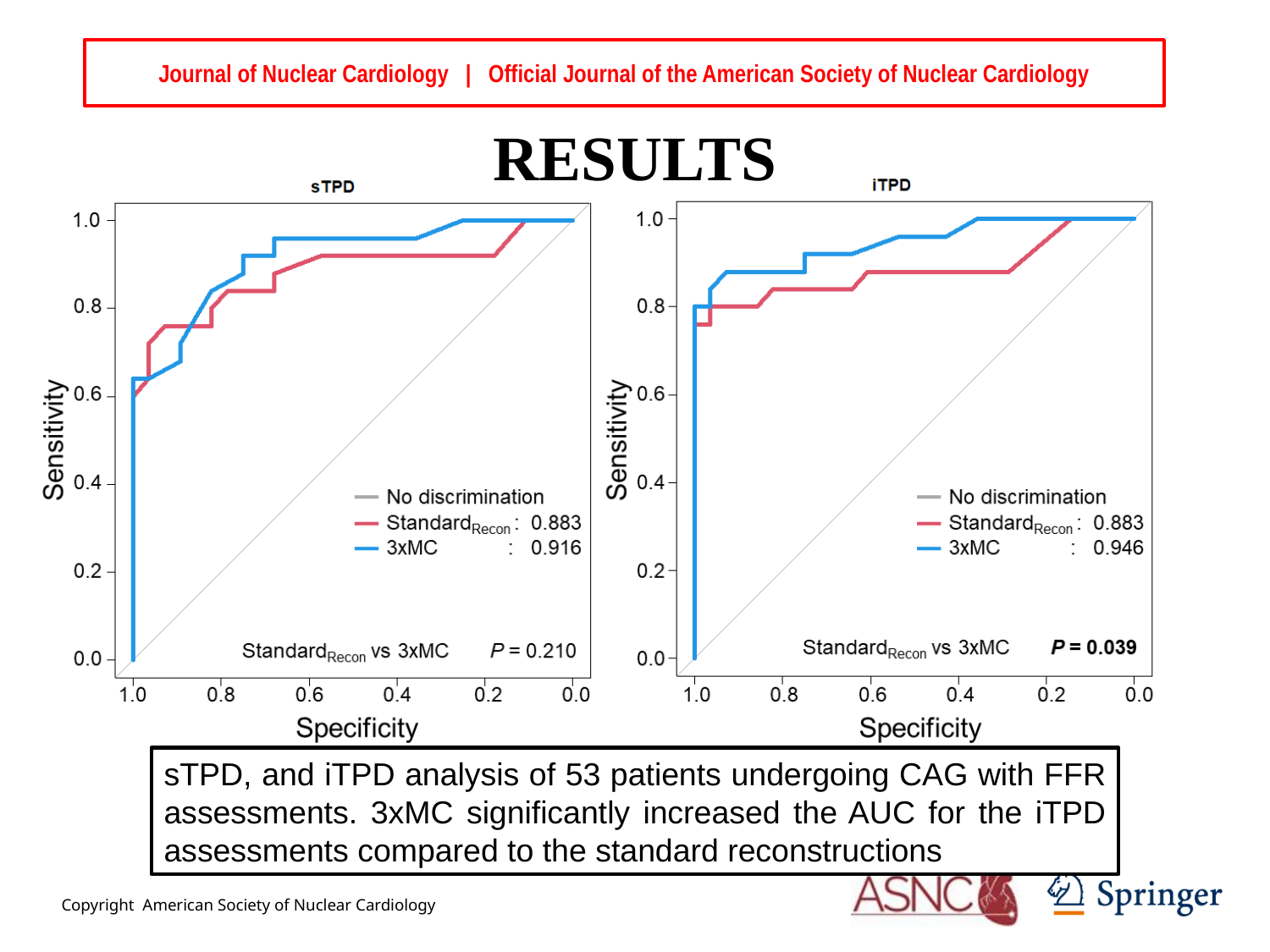

Journal of Nuclear Cardiology | Official Journal of the American Society of Nuclear Cardiology
# RESULTS
sTPD, and iTPD analysis of 53 patients undergoing CAG with FFR assessments. 3xMC significantly increased the AUC for the iTPD assessments compared to the standard reconstructions
Copyright American Society of Nuclear Cardiology

## Slide 5
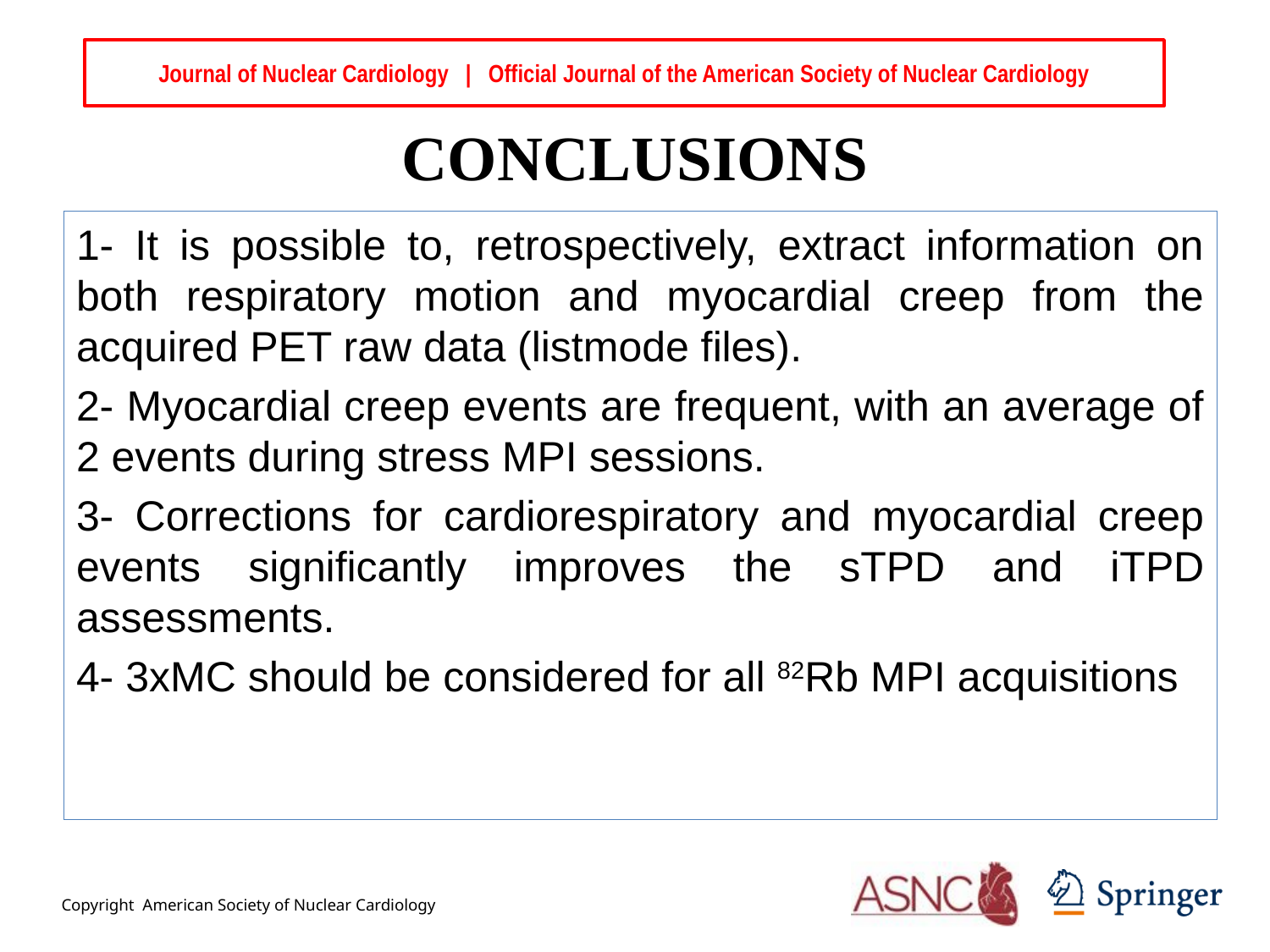

Journal of Nuclear Cardiology | Official Journal of the American Society of Nuclear Cardiology
# CONCLUSIONS
1- It is possible to, retrospectively, extract information on both respiratory motion and myocardial creep from the acquired PET raw data (listmode files).
2- Myocardial creep events are frequent, with an average of 2 events during stress MPI sessions.
3- Corrections for cardiorespiratory and myocardial creep events significantly improves the sTPD and iTPD assessments.
4- 3xMC should be considered for all 82Rb MPI acquisitions
Copyright American Society of Nuclear Cardiology
